# Influence of Dental Education on Esthetics Self-Perception and Shade Selection

**DOI:** 10.3390/ijerph191811547

**Published:** 2022-09-14

**Authors:** Passent Ellakany, Shaimaa M. Fouda, Maram A. AlGhamdi, Nourhan M. Aly

**Affiliations:** 1 Department of Substitutive Dental Sciences, College of Dentistry, Imam Abdulrahman Bin Faisal University, Dammam 32210, Saudi Arabia; 2 Department of Pediatric Dentistry and Dental Public Health, Faculty of Dentistry, Alexandria University, Alexandria 21527, Egypt

**Keywords:** esthetics, self-perception, digital shade, spectrophotometer, dental education, students

## Abstract

A discrepancy is encountered between the esthetic self-perception and the actual need for treatment. The aim was to determine the influence of dental education on the patients’ attitude, esthetic self-perceptions, and shade selection. This cross-sectional study asked participants to select the perceived shade of their incisors using a shade guide, and to complete a self-administered questionnaire assessing their attitude regarding teeth shade. The color coordinates (L*, a*, and b*) of their actual shades were recorded clinically, using a digital spectrophotometer. A Pearson correlation assessed the relation between the perceived and actual color coordinates. A linear regression assessed the association between the attitude towards the esthetic self-perception, background factors, and actual shades. A clinical shade selection was done digitally for 536 participants, comprising 40.1% preclinical dental students, 37.3% clinical dental students, and 22.6% non-dental participants. The perceived and actual a* and b* values were significantly correlated in the preclinical students, and L* and b* were correlated in the clinical students. Those who had not received any dental education showed better attitude scores than the clinical students. The color-matching skills were improved by education; therefore, this emphasizes the importance of teaching color selection in dental schools. Dental esthetic self-perception is also improved by increasing knowledge and skills through exposure to a variety of comprehensive dental cases.

## 1. Introduction

Esthetic dental treatment has become a prime concern for all individuals, regardless of their education and culture [1,2]. The satisfaction with one’s dental appearance increases individuals’ self-esteem, which subsequently affects their ‘attitude, behavior, social relations, and even job opportunities’ [3,4]. In order to meet the patients’ esthetic needs and expectations, dental professionals are required to be competent and skillful in shade selection, and they should understand the factors that influence patients’ intentions to seek esthetic dental treatments. Television presenters and artists have an increased tendency to seek more cosmetic dental treatments, such as Hollywood smiles, and this has an influence on patients and makes them more eager to achieve the same esthetic outcome [5,6,7,8]. Therefore, dentists should establish a proper treatment plan that matches the patients’ esthetic needs and functional demands [4,9].

Attractive esthetic smiles depend on the harmony between several factors, such as the tooth color, shape, dimensions, and alignment, in addition to the lip line, buccal corridor, and midline position, in relation to the face [4]. Also, symmetry is a crucial factor that contributes to creating an esthetic smile, particularly in the case of restoring the maxillary incisors using esthetic restorations such as composite resins, laminate veneers, and single crowns [10]. Furthermore, the direct restorative procedures of the anterior teeth, such as composite restorations, has become more demanding for dental operators due to the ease of application and affordability for the patients [11,12]. Although composite application presents predictable color-matching results, still, esthetic failures may occur during the clinical service because of the color instability of the resin-based composites [13].

The perception of esthetic smiles might vary according to demographic factors that include age, gender, and education. Also, personal experiences, cultural and geographic variations might widely affect the esthetic perception of an acceptable smile [1,7,14,15], since dental esthetics is subjective, unlike other objective dental procedures such as root canal treatment and oral surgeries [5]. This variation in the smile perception was documented in various populations such as the United States, Canada, Saudi Arabia, and India [16,17,18,19]. It was reported in the literature that an acceptable dental appearance depends mainly on matching tooth color, which is an essential requirement for a reputable job in certain fields [4,20]. The educational background is believed to affect the smile perception of individuals, as dental education is associated with a better esthetic perception than individuals of other educational backgrounds [21]. Despite this, the esthetic perception of dental students fluctuates, as it depends on the educational level of the students [22], where younger students might have a different perception than older students, and this might be related to the higher dental knowledge and exposure to dental patients in the clinical dental years [22,23].

Visual shade selection is considered the most common method used in dentistry to achieve an acceptable esthetic restoration. Several color difference equations are used in providing quantitative readings of the perceived color difference when comparing two objects in dental experiments, where ∆E* represents the difference in color measurements. For example, high ∆E* readings are an indicator of big differences in color between the two objects, which can be easily perceived by human vision. The color difference was also assessed by two main thresholds, named the perceptibility threshold (PT) and the acceptability threshold (AT). A 50:50% PT is defined as noticing a color difference between the two specimens by only half of the viewers, while 50:50% AT means that only half of the viewers noticed an unacceptable color difference between both specimens, that requires refabricating the esthetic restoration [24].

The discrepancy encountered between the esthetic self-perception and the actual need for dental esthetic treatment raises the attention of the investigator to explore how the perception of dental esthetics is observed differently within the same society, and the way it affects the psychosocial well-being of individuals [4].

Limited studies in literature were found assessing esthetic self-perception among dental students, but no previous studies were carried out to evaluate the esthetic perception of dental students compared to the general population in Saudi Arabia. Thus, the aim of the current study was to determine the influence of dental education on the participants’ dental esthetic attitude, esthetic self-perceptions, and shade selection. The hypothesis states that the dental esthetic attitude and esthetic self-perceptions of dental students would differ from individuals with no dental education.

## 2. Materials and Methods

### 2.1. Study Design

The current cross-sectional study was carried out from September 2021 to February 2022. The study was approved by the institutional review board of Imam Abdulrahman Bin Faisal University, Dammam, Saudi Arabia (IRB-2022-02-284) and in accordance with the Declaration of Helsinki. The study assessed the influence of dental education on the esthetic self-perception and shade selection.

### 2.2. Participants and Sampling

A convenience sample of Saudi patients and dental students was recruited from the College of Dentistry of Imam Abdulrahman Bin Faisal University, Dammam, Saudi Arabia. The participants included in the current study were individuals having sound, natural maxillary central incisors, free of dental caries or restorations and any systemic diseases. The sample size calculation was estimated assuming a 95% confidence level and 5% margin of error. Based on Dudea et al. [7], 39.07% of the participants indicated an abnormal self-perceived dental shade. The minimum required sample size was calculated to be 366, and the total enrolled number of participants in this study was 536, grouped into (1) participants from the general population, (2) pre-clinical dental students, and (3) clinical dental students. The participants from the general population were those who had not received any dental education or training. The preclinical dental students group included students from the second and third years of dental education, who had acquired basic dental knowledge and practiced dental skills in phantom laboratories, while the clinical dental students group included students from the fourth to the sixth year of dental education. Fourth-year students initiate their clinical training in the dental hospital, followed by carrying out comprehensive dental treatments of different difficulty levels in the clinics of the dental college by the fifth and sixth years.

### 2.3. Questionnaire and Data Collection

The study used a self-administered paper-based questionnaire composed of four sections [1]. The first section included the sociodemographic variables of the participants (age, gender, educational level, and dental year (if applicable)). The second section included nine questions assessing the esthetic attitude of the participants; the third section included the esthetic self-perception through the self-selection of the central incisors’ shade, and the fourth section included the actual digital shade selection of the same teeth of the participants by a trained operator (P.E.). The questionnaire was developed in the Arabic language to be suitable for all the participants living in Saudi Arabia [1]. A validation of the questionnaire was done, and the overall content validity index of the tool was 0.83. The questionnaire was preceded by a brief introduction about the study team and objectives, the time needed to complete the questionnaire (≈5 min), ensuring that participation was voluntary, and the assurance that the confidentiality of the responses was secured.

The attitude towards the tooth shade was assessed using nine questions on a five-point Likert scale, ranging from 0 “not at all” to 5 “completely”. The questions assessed the impact of the esthetic appearance and tooth shade on participants, including being happier, looking younger, looking good, getting a good job opportunity, and how important to look like the people in TV advertisements. The scoring was performed by calculating the average of the nine questions to range from zero to five, with the higher scores indicating a better attitude.

The self-perception of the tooth shade was done by a shade tab from the Vitapan Classical Shade Guide (Vita-Zahnfabrik, Bad Säckingen, Germany) under natural daylight in the dental clinic (perceived shade). The Vitapan Classical Shade Guide is divided into four groups, depending on the hue (A, B, C, D), where each group has four shade tabs arranged ascendingly according to the chroma intensity (1–4). The higher the chroma of the selected shade tab, (for example A4), the lower the value would be for the same shade tab [25]. The actual shade selection was determined digitally after performing a clinical examination, and the removal of any plaque or calculus on the labial surface of the central incisors by the operator under the same lightening conditions. A digital spectrophotometer (Crystaleye; Olympus, Tokyo, Japan) characterized the color by showing the tooth shade with a real image on a computer screen of the same imaging analysis software (Crystaleye application master; Olympus, Tokyo, Japan), which showed the shape and color of the examined tooth for a better shade selection. The spectrophotometer was calibrated and utilized, according to the manufacturer’s instructions, to provide the digital shade readings of the middle third of the labial surface of the central incisors in the form of L, a, and b, according to the CIELAB (Commission Internationale de l’Eclairage), where “L” represents the degree of lightness of the tooth, “a” represents the red to green color of the tooth, and “b” represents the yellow to green color.

### 2.4. Statistical Analysis

The data were analyzed using the IBM SPSS for Windows (Version 23.0, IBM Corp., Armonk, NY, USA), and the significance was inferred at *p* value < 0.05. The normality was checked for all the variables using descriptive statistics, plots, and normality tests. All the variables showed normal distribution, so the data were presented as the means and standard deviations (SD). The frequencies and percentages were calculated for the qualitative variables. The esthetic self-perceived shade by the participants (using the Vitapan Classical Shade Guide) was transformed into L*, a*, and b* values, based on the values reported by Tashkandi [26] as he proved a great consistency in the color parameters of the Vitapan Classical Shade Guide, and so translated the 16 shade guide designations into the L*, a*, and b* values. The digital L*, a*, and b* values (actual shades) were averaged across the three regions per tooth, and the values of both central incisors were averaged to give the person’s average. A Pearson correlation was performed to assess the relation between the perceived and actual digital color coordinates (L*, a*, and b*). A linear regression was used to assess the association between the attitude towards the esthetics self-perceived appearance, background factors and the actual digital tooth shade. The regression coefficients (B) and 95% confidence intervals (CI) were calculated.

## 3. Results

**Figure 1:**
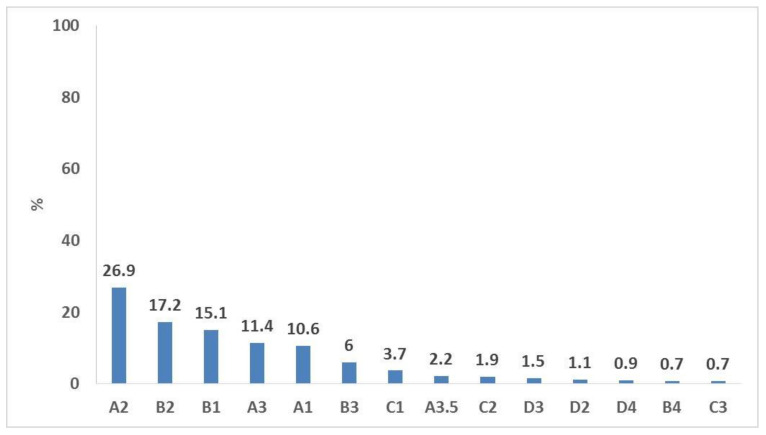
Self-perceived shades among the study participants.

Six hundred individuals were invited to participate, but only 536 agreed to participate (89.3% response rate). Table 1 shows that the mean ± SD age was 24.48 ± 8.36, with 40.1% preclinical students and 37.3% clinical students. Shades A2, B2, B1, A3, and A1 were selected by 81.2% of the participants (Figure 1). The mean ± SD perceived L*, a*, and b* values = 59.62 ± 1.95, 0.40 ± 0.71, and 8.69 ± 1.93, respectively. The mean ± SD L* value = 67.44 ± 5.29, a* value = 3.46 ± 2.08, and b* value = 20.08 ± 3.14.

**Figure 2:**
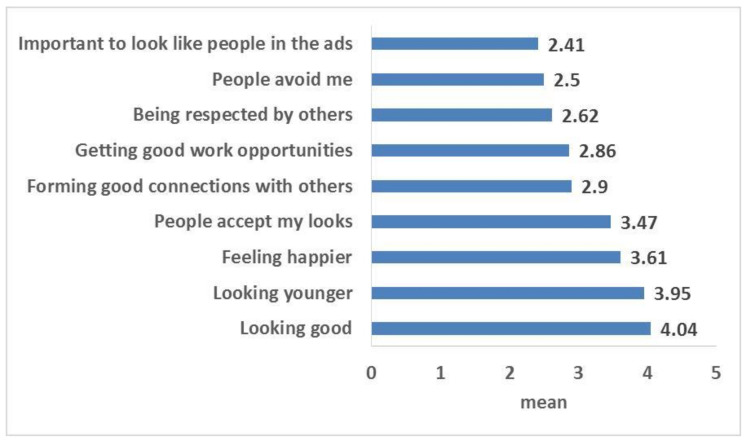
Attitude towards self-perceived tooth shade among the study participants (5 = completely agree).

Figure 2 shows that the participants agreed that tooth shade affects whether they look good, look younger, feel happier, and also affects people’s impression of their looks (mean = 4.04, 3.95, 3.61, and 3.47, respectively). They agreed less on other items, including being respected by others and getting good work opportunities (mean = 2.62 and 2.86).

Table 2 represents the correlation between the self-perceived and actual digital shade. On average, the perceived L* and b* values were significantly correlated with the actual L* and b* values (r = 0.16 and 0.17, *p* < 0.001). In the males, there was a significant correlation between the perceived and actual L*, a*, and b* values (*p* < 0.05), but the correlation was non-significant for the females. Regarding dental education, the self-perceived and actual L*, a*, and b* values were not significantly correlated in those who had not received any dental education. However, the a* and b* values were significantly correlated in the pre-clinical students (r = 0.14, *p* = 0.04 and r = 0.20, *p* = 0.003), and L* and b* were significantly correlated in the case of the clinical dental students (r = 0.34 and 0.32, *p* < 0.001).

Table 3 represents the association between the dental esthetic attitude with the self-perceived tooth shade and different background factors. In the univariate regression (unadjusted model), an older age was significantly associated with a better attitude score (B = 0.008, *p* = 0.04), while the perceived b* was associated with lower attitude scores (B = −0.04, *p* = 0.03). Also, the males (versus the females) who had not received any dental education and preclinical students (versus clinical students), had significantly better attitude scores. In the adjusted model, the males showed better attitude scores than the females (B = 0.18, *p* = 0.009). Those who had not received any dental education also showed better attitude scores than the clinical dental students (B = 0.37, *p* = 0.008). The adjusted R^2^ of the model = 0.03.

## 4. Discussion

The present study evaluates the influence of dental education on the esthetic attitude, esthetic self-perception, and shade selection. The study hypothesis was partly accepted, as the esthetic attitude and esthetic self-perceptions of the clinical dental students differed significantly from the preclinical dental students and individuals who had not received any dental education.

Two methods were used in this study to determine the tooth shade, the Vitapan Classical Shade Guide and a digital spectrophotometer. The Vitapan Classical Shade Guide was used in detecting the self-perceived shade by the participants, as this guide is characterized by its high consistency and simplicity of use [1,6]. However, the actual shade was detected directly from the participants’ oral cavities by the digital spectrophotometer to provide standardized accurate readings, excluding any human variables and the lighting of the surrounding environment, which provides a highly valid method in assessing the tooth shade of all the participants [1,26,27].

Most participants in the current study selected the shades A2, B2, B1, A3, and A1. This agrees with other studies. Shade A3 was the most selected by the Sudanese participants in Elamin et al.’s study [10]. Also, shades A1 and A2 were the most chosen shades among Indian participants, as reported by Roderigues et al. [28].

The participants in the current study stated that the tooth shade affects their looks and other people’s perception of their appearance. Moreover, they agreed that their tooth shade could make them look younger and feel happier. Similarly, previous studies reported a correlation between patient satisfaction with their appearance and the shade of their teeth or dental prosthesis [29,30].

Females are usually more concerned with their appearance and, consequently, the shape of their teeth than males [4,31,32]. In the current study, the males were more satisfied with their teeth shades than the females. This agrees with other studies that reported higher satisfaction with the perceived dental appearance among the males than the females, who showed higher dental concerns and oral demands when compared to the males [31,32]. In line with the present results, Ellakany et al. [4] reported lower dental self-confidence among adolescent females in Saudi Arabia compared to males.

The present results reveal an association between the esthetic self-perception and dental education. The participants who had not received any dental education, and the pre-clinical dental students, were more satisfied with their self-perceived dental esthetics than the clinical dental students. The reason for that could be the dental education and training, which made the clinical students aware of the acceptable and unacceptable dental esthetics [33,34]. Their knowledge about the ideal shape, shade, and alignment of teeth might make them more aware of the perfect dental look.

Moreover, the results show a positive correlation between the perceived and actual color coordinates [L*, a*, and b* values] among the dental students, unlike those who had not received any dental education. Supporting the current results, Samorodnitzky-Naveh et al. [35] reported that only 18.7% of the participants included in their study perceived a similar shade selection as that selected by the clinician. Previous studies reported that dental education and training improved the students’ shade-matching clinical skills [33,34,36]. Similarly, Ristic et al. [37] reported that the students who received training on shade selection had improved shade-matching skills compared to those who did not receive any training or education on color selection. The preclinical students included in this study had not received any lectures on shade selection in their preclinical fixed prosthodontics courses, unlike the clinical students, who had received two lectures per year, in addition to practicing shade selection in the 4 h weekly clinical sessions of the restorative, fixed prosthodontics, and comprehensive clinical courses.

There was a significant correlation between the perceived and actual color coordinates among the males, but not the females. Previous studies showed different results regarding the influence of gender on the shade selection. Some studies have reported non-significant differences between males and females [38,39,40], while other studies stated that gender affects the shade selection [41,42]. In agreement with the present results, studies show that males have better color perception than females [42,43]. On the contrary, Alfouzan et al. [44] and Samra et al. [38] reported a non-significant difference between males and females, in relation to shade-matching skills, before and after training on shade selection. The differences between the present findings and other studies might be due to the differences in the methods used in the shade selection, in addition to the differences in the sample size and the percentage of males to females.

The strengths of the present study were the inclusion of a larger number of participants, compared to previous studies [23,33,38,43], and including the same number of males and females, which increases the credibility of the findings. In addition, clinical examinations were performed on the participants using a digital spectrophotometer, in order to obtain standardized shade readings to provide standardized measurements, excluding any human variations or eye fatigue. Despite this, the study has some limitations. Among these are the quite low correlation coefficients, which indicate a weak correlation, despite being significant. Similarly, the model’s adjusted R^2^ was quite low, indicating that other explanatory factors need to be further explored. Another limitation is the sampling strategy, in which a convenience sample from one dental college was recruited. Also, the study can’t be generalized to other populations since it is confined to Saudi populations, who have different norms and cultural beliefs than other populations. Further studies are needed to assess the difference in the esthetic perceptions in different Saudi dental colleges, in addition to adding dental colleges from other countries.

## 5. Conclusions

Based on the findings of our study, it can be concluded that the color-matching skills and esthetic self-perception were improved by dental education in Saudi colleges; therefore, it is essential to emphasize the importance of teaching color selection in Saudi dental schools. Dental esthetic self-perception is improved by increasing dental knowledge and skills through exposure to a variety of comprehensive dental cases in the clinical dental sessions. The didactic part of dental esthetics taught to students is not enough to gain better esthetic perception, so it needs to be enhanced with clinical training.

## Figures and Tables

**Table 1:** Sample characteristics (*n* = 536).

**Age**	**Mean ± SD**	24.48 ± 8.36
**Gender: *n* (%)**	**Males**	268 (50%)
	**Females**	268 (50%)
**Dental education: *n* (%)**	**No dental education**	121 (22.6%)
	**Pre-clinical students**	215 (40.1%)
	**Clinical students**	200 (37.3%)
Perceived L*	**Mean ± SD**	59.62 ± 1.95
Perceived a*	**Mean ± SD**	0.40 ± 0.71
Perceived b*	**Mean ± SD**	8.69 ± 1.93
L*	**Mean ± SD**	67.44 ± 5.29
a*	**Mean ± SD**	3.46 ± 2.08
b*	**Mean ± SD**	20.08 ± 3.14
**Self-perceived attitude towards esthetics**	**Mean ± SD**	3.15 ± 0.79

**Table 2:** Correlation between perceived and actual digital shade (color coordinates).

		L*	a*	b*
**Gender**	**Males**	r = 0.23	r = 0.19	r = 0.22
		*p* < 0.001 *	*p* = 0.002 *	*p* < 0.001 *
	**Females**	r = 0.08	r = −0.05	r = 0.12
		*p* = 0.17	*p* = 0.40	*p* = 0.049 *
**Dental education**	**No dental education**	r = −0.09	r = −0.03	r = 0.04
		*p* = 0.33	*p* = 0.76	*p* = 0.65
	**Pre-clinical students**	r = 0.09	r = 0.14	r = 0.20
		*p* = 0.17	*p* = 0.04 *	*p* = 0.003 *
	**Clinical students**	r = 0.34	r = 0.07	r = 0.32
		*p* < 0.001 *	*p* = 0.32	*p* < 0.001 *
**Total**		r = 0.16	r = 0.04	r = 0.17
		*p* < 0.001 *	*p* = 0.32	*p* < 0.001 *

r = Pearson correlation coefficient; * statistically significant at *p* value < 0.05.

**Table 3:** Association between dental esthetic attitude, background factors, and self-perceived tooth shade.

		Unadjusted Model		Adjusted Model	
		B (95% CI)	*p* Value	B (95% CI)	*p* Value
**Age**		0.008 (0.004, 0.02)	0.04 *	−0.0004 (−0.01, 0.01)	0.95
**Gender (males vs. females)**		0.16 (0.02, 0.29)	0.02 *	0.18 (0.05, 0.32)	0.009 *
**Dental education**	**No dental education**	0.34 (0.17, 0.52)	<0.001 *	0.37 (0.10, 0.64)	0.008 *
	**Pre-clinical students**	0.17 (0.02, 0.32)	0.03 *	0.15 (−0.01, 0.32)	0.07
	**Clinical students**	Reference category			
Perceived L*		0.005 (−0.03, 0.04)	0.79	0.01 (−0.03, 0.05)	0.54
Perceived a*		−0.08 (−0.17, 0.02)	0.12	0.05 (−0.12, 0.22)	0.59
Perceived b*		−0.04 (−0.07, −0.004)	0.03 *	−0.04 (−0.10, 0.02)	0.19
L*		−0.009 (−0.02, 0.004)	0.19	−0.003 (−0.02, 0.01)	0.70
a*		−0.01 (−0.04, 0.02)	0.49	−0.03 (−0.07, 0.02)	0.20
b*		−0.003 (−0.02, 0.02)	0.81	0.005 (−0.02, 0.03)	0.73

Model F: 3.05, * *p* = 0.002, Adjusted R^2^ = 0.03.

## Data Availability

Data is available upon request from the corresponding author.
